# Enhancing Clinical Documentation: The Effect of Structured Templates on Follow-Up Notes in a Low-Resource Hospital Setting

**DOI:** 10.7759/cureus.88510

**Published:** 2025-07-22

**Authors:** Elaf Abdelrahman, Abdelrahman Abdelrahim, Thekra Mohamed, Maram Babikir, Nirmeen Ahmed, Hanan Gandour, Noon Ahmed, Ahmed Elsayed, Ola Ahmed, Ahmed Saeed, Mohamed Alsayed, Amro Mohammed, Hebat Allah N Fadhl, Altayib Abdalrahman, Nassma Ibrahim, Mustafa Mohamed

**Affiliations:** 1 Internal Medicine, Al-Neelain University, Khartoum, SDN; 2 Internal Medicine, Almanagil Teaching Hospital, Almanagil, SDN; 3 Medical Education, Al-Neelain University, Khartoum, SDN; 4 General Practice, University of Medical Sciences and Technology, Khartoum, SDN; 5 General Practice, Al-Neelain University, Khartoum, SDN; 6 General Surgery, Al-Neelain University, Khartoum, SDN; 7 Medical Education, Faculty of Medicine and Health Sciences, Aden University, Aden, YEM; 8 General Practice, Hasahesa Teaching Hospital, Khartoum, SDN; 9 Medicine, Al-Neelain University, Khartoum, SDN; 10 Internal Medicine, Prince Othman Digna Teaching Hospital, Port Sudan, SDN

**Keywords:** clinical documentation, follow-up notes, hasahesa hospital, medical audit, nice guidelines, quality improvement, structured templates, sudan healthcare

## Abstract

Background: Accurate and structured clinical documentation is essential for delivering high-quality patient care. While structured documentation tools have been increasingly adopted in well-resourced healthcare settings, many low-resource environments, such as Sudan, continue to rely predominantly on traditional, unstructured methods. This study aimed to evaluate the effectiveness of implementing structured documentation practices in improving the quality of clinical notes at Hasahesa Hospital.

Methodology: A mixed retrospective and prospective audit was conducted in two cycles at Hasahesa Hospital. The first cycle involved a retrospective review of 50 clinical records from July 2023, assessed using a standardized checklist developed based on National Institute for Health and Care Excellence (NICE) guidelines. Following a targeted training intervention, the second cycle prospectively evaluated another 50 clinical records from September 2023. Documentation quality was measured by compliance with ten key parameters. Data analysis was performed using Microsoft Excel, with compliance rates compared between the two cycles.

Results: There were significant improvements in documentation quality following the intervention, with overall compliance increased from 38.2% to 87.2% (*P* < 0.001). Notable enhancements were seen in documentation of date and time (increased by 63%), chief complaints (60%), and history of present illness (45%). Documentation of current medications improved from 15% to 79%, while vital signs and physical examination recording increased by 18% and 40%, respectively. Documentation of laboratory results showed the greatest improvement, increasing by 75%. These results highlight the positive impact of structured documentation and targeted training on clinical record-keeping practices.

Conclusions: The introduction of structured documentation significantly improved the completeness and quality of follow-up clinical notes at Hasahesa Hospital. Despite these improvements, areas such as history of present illness, past medical history, and current medications require continued attention to achieve optimal compliance. These findings emphasize the necessity of ongoing staff training and the use of standardized templates to maintain and further enhance documentation standards, thereby optimizing patient care. Future audits should also explore expanding structured documentation practices across other hospital departments and settings.

## Introduction

Clinical notes are essential for documenting the care a patient receives, enabling healthcare providers to track treatment decisions, monitor the patient’s condition over time, and record disease progression. Accurate clinical documentation also supports research and education by detailing the patient’s status and treatment plan. Ensuring that clinical documentation meets the informational needs of both clinicians and patients is crucial, as clinical notes are a key component of medical records and significantly impact the quality of patient care. Healthcare providers must maintain accurate and up-to-date clinical records to deliver the best possible treatment [1].

Content Importation Technologies (CITs) refer to tools such as templates, macros, and auto-population features designed to improve documentation efficiency and accuracy. These tools are particularly valuable in resource-constrained healthcare settings, where time and personnel may be limited. Electronic Health Records (EHRs) commonly incorporate these functionalities, providing benefits such as increased consistency and faster documentation processes [2]. This project utilizes such technologies to enhance the quality and completeness of clinical documentation.

Improving documentation efficiency while maintaining accuracy is essential. Greater standardization of documentation is necessary because variability among physicians in EHR documentation hinders the safe and effective use of these systems [3]. Structured documentation, defined as an organized and standardized recording, can sometimes make note interpretation more challenging. Rosenbloom et al. [4] explored this balance between structured and flexible narrative documentation and concluded that healthcare personnel should be able to document patient care in a way that aligns with their clinical workflow while ensuring that essential content is captured. This workflow flexibility can be achieved using standardized checklists, templates, or hybrid models that combine structured fields with narrative sections. Structured documentation is preferred for data reuse, whereas narrative documentation may be appropriate when restating information is unnecessary.

Structured documentation is increasingly recognized for its role in supporting data reuse and improving the consistency of clinical notes [5]. However, the primary goal of clinical note writing remains to facilitate high-quality patient care [6]. While studies have suggested potential benefits and challenges related to the transition from free-form, unstructured notes to standardized documentation, our study did not assess provider time or feedback regarding this change.

In paper-based institutions like Hasahesa Hospital, where digital technologies are lacking, alternative approaches are necessary to achieve similar improvements in documentation quality. Organizations such as the National Institute for Health and Care Excellence (NICE) have developed standards and recommendations that can be effectively implemented through structured templates and standardized checklists. Even without the technical benefits of CITs, these manual methods improve the quality of clinical notes by promoting thorough and consistent documentation [1].

## Materials and methods

This study, which combined both retrospective and prospective audits, was carried out within the Internal Medicine Department of Hasahesa Teaching Hospital, a large medical facility in Gezira state in Sudan, equipped with over 150 beds. The study obtained ethical approval from the Institutional Review Board of the hospital. The study's primary focus was to evaluate the standard of follow-up clinical documentation through the use of a pre-designed template, adhering to guidelines from the NICE (Table 1) [1]. The aim was to identify any shortcomings in the existing documentation practices and apply structured interventions to improve the format and content of clinical notes.

**Table 1:** The follow-up checklist criteria as recommended by NICE guidelines. Essential parameters evaluated in the follow-up clinical notes checklist, adapted from NICE guidelines, are designed to ensure thorough, standardized, and high-quality documentation that supports effective patient management and continuity of care. Source: [1]. NICE, National Institute for Health and Care Excellence

Parameters checklist
Date and time
Chief complaint (CC)
History of present illness (HPI)
Past medical history (PMH)
Current medications
Vital signs
Physical examination
Laboratory results
Diagnosis
New plan of management

Aim and objectives

The purpose of this audit was to evaluate the Internal Medicine Department’s follow-up clinical documentation practices in comparison to standard benchmarks and identify opportunities for improvement. Ultimately, the aim was to enhance clinical decision-making and patient care outcomes by making follow-up notes clearer, more comprehensive, and accurate.

Audit area/population

The audit focused on follow-up clinical records from the Internal Medicine Department at Hasahesa Hospital. Two evaluation phases were conducted: a four-day retrospective review in January 2025 and a prospective assessment in March 2025. Inclusion criteria were all follow-up clinical notes documented for adult patients attending the department during these periods. Notes were excluded if they were incomplete, illegible, or related to initial consultations rather than follow-up visits. The primary goal of both stages was to assess adherence to the NICE recommendations for recording follow-up treatment and identify areas for quality improvement.

Sample size and sampling technique

The sample size of 50 clinical records per audit cycle was chosen based on feasibility and resource availability during the designated four-day review periods. This approach is consistent with previous audits in similar settings. While no formal power calculation was conducted, this sample size was adequate to observe significant changes in documentation quality following the intervention.

A simple random sampling method was employed in both cycles to select the records. This approach ensured that each follow-up clinical note within the Internal Medicine Department had an equal probability of inclusion, thereby enhancing the representativeness of the sample relative to the department’s overall patient population. The sampled cases reflected a diverse cross-section of patients, capturing variations in demographics, diagnoses, and clinical complexity. This sampling strategy aimed to minimize selection bias and provide a robust, generalizable assessment of documentation quality across routine follow-up encounters in the department.

Data collection and analysis

Data collection was performed by trained medical professionals who meticulously reviewed follow-up clinical notes, employing a standardized checklist designed based on the NICE guidelines. This checklist evaluated 10 critical elements of documentation, including the presence of date and time, clinical findings, updated diagnoses, treatment modifications, medication lists, laboratory results, and planned follow-up instructions. The aim was to assess both the completeness and accuracy of the recorded information.

All data collected during both cycles were entered into Microsoft Excel 2016 (Version 16.0; Microsoft Corporation, Redmond, WA) for organization and statistical analysis. The primary analytical method employed was the Chi-square (χ²) test to compare categorical variables, specifically compliance rates for each documentation parameter between the two audit cycles. The threshold for statistical significance was set at *P* < 0.05. This inferential statistical approach allowed the detection of significant improvements or ongoing deficiencies in documentation quality attributable to the interventions applied.

Overall compliance was calculated by dividing the total number of documented parameters across all notes by the total number of expected parameters (i.e., number of notes × 10 parameters), then multiplying by 100 to obtain a percentage. The full checklist is provided in the Appendix.

Audit cycles

First Cycle: Pre-intervention

The initial audit cycle was conducted over a defined period in January 2025, aiming to assess the existing state of follow-up clinical note documentation at Hasahesa Hospital. This baseline assessment revealed notable deficiencies in completeness and consistency. Commonly missing or inadequately recorded information included the date and time of the patient encounter, detailed clinical findings, updates to diagnoses, medication lists, and clear follow-up plans. Such gaps not only compromise patient safety and clinical continuity but also weaken medico-legal safeguards.

Intervention Phase: Implementation

**Figure 1:**
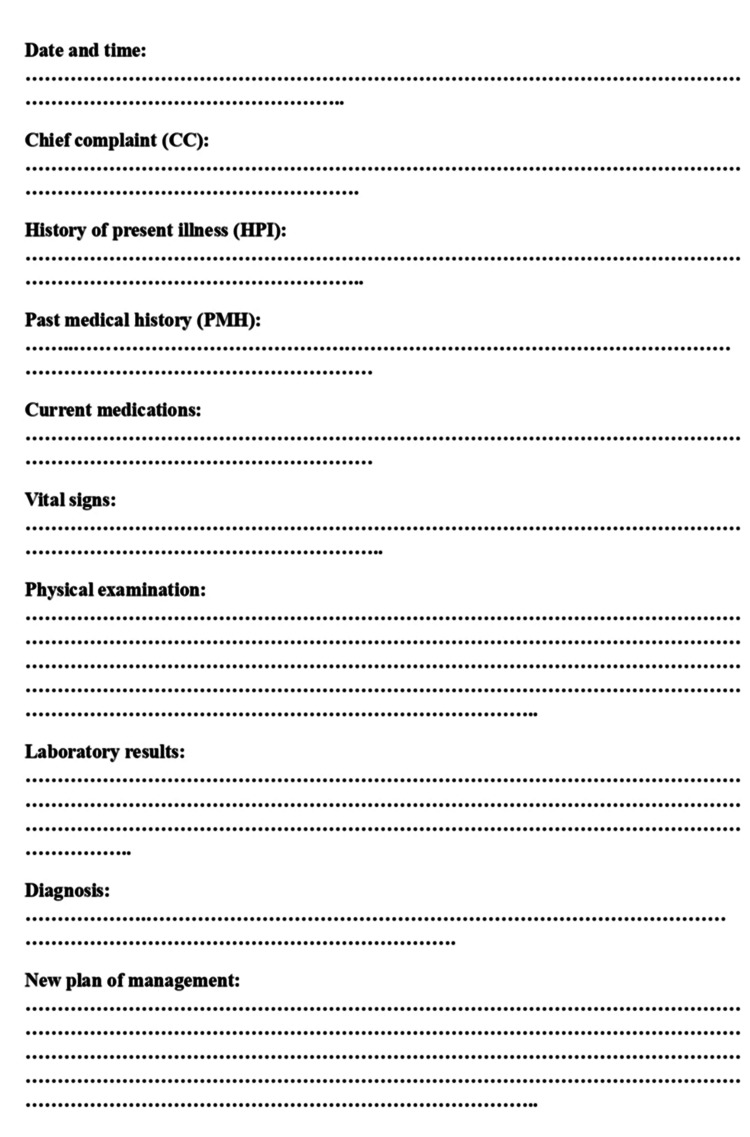
Checklist performa for follow-up medical documentation. This figure illustrates the structured checklist used for assessing follow-up medical documentation quality during the audit. The checklist comprised 10 essential items: Date and Time, Chief Complaint (CC), History of Present Illness (HPI), Past Medical History (PMH), Current Medications, Vital Signs, Physical Examination, Laboratory Results, Diagnosis, and New Plan of Management. Each item was evaluated for presence and completeness in the medical notes. Image credit: Audit Team, Department of Medicine, Al-Hasahesa Teaching Hospital.

In response to the gaps identified during the initial cycle, a multifaceted intervention strategy was developed and implemented. A standardized follow-up note proforma (Figure 1), aligned with NICE clinical documentation guidelines, was introduced to provide a clear and consistent structure for note-taking. To ensure effective uptake of the new proforma, comprehensive educational sessions and training workshops were delivered to clinical staff, emphasizing the importance of thorough documentation for patient safety, communication, and medicolegal considerations.

To reinforce these educational efforts, visual reminders summarizing key documentation elements were strategically placed within clinical areas, including wards and consultation rooms. Additionally, responsibilities for completing follow-up notes were clearly assigned to designated staff members, and regular feedback sessions were established to monitor progress, address challenges, and maintain accountability.

Second Cycle: Post-intervention

The second audit cycle, conducted prospectively in March 2025, evaluated the impact of the implemented interventions. Several documentation parameters demonstrated marked improvement compared to the first cycle. Details of compliance rates and statistical significance are presented in the Results section.

To sustain these improvements, periodic audits and continued staff education have been planned to reinforce structured documentation practices and promote long-term quality enhancement.

Ethical considerations

Ethical clearance for this audit was secured from the Institutional Review Board (IRB) of Hasahesa Hospital, referenced under IRB number HA2025/011.

## Results

A total of 100 follow-up clinical notes were reviewed - 50 during the first (retrospective) audit cycle in January 2025 and 50 during the second (prospective) cycle in March 2025. Baseline findings revealed notable deficiencies in documentation, with an overall compliance rate of 38.2% across the 10 evaluated parameters. Following the intervention, which included the introduction of a structured proforma and staff training, overall documentation compliance improved significantly to 87.2%.

Several parameters demonstrated substantial gains. Documentation of the date and time of the clinical encounter improved from 17 (34%) to 45 (90%) notes (χ² = 30.94, *P* < 0.001), while inclusion of the chief complaint increased from 15 (30%) to 44 (88%) notes (χ² = 32.41, *P* < 0.001). The number of records documenting the history of present illness rose from 15 (30%) to 38 (76%) (χ² = 19.43, *P* < 0.001). Past medical history was recorded in only 10 (20%) notes in the first cycle, compared to 40 (80%) in the second (χ² = 33.64, *P* < 0.001). Documentation of current medications showed a similar trend, increasing from 8 (16%) to 40 (80%) (χ² = 38.50, *P* < 0.001).

Clinical examination parameters also showed marked improvement. Vital signs, which were initially documented in 40 (80%) of the records, increased to 49 (98%) (χ² = 6.54, *P* = 0.011). Physical examination findings improved from 27 (54%) to 47 (94%) (χ² = 18.76, *P* < 0.001). Laboratory results demonstrated the most significant change, rising from 8 (16%) to 45 (90%) (χ² = 52.03, *P* < 0.001). The number of records, including a new or updated provisional diagnosis, increased from 10 (20%) to 45 (90%) (χ² = 46.71, *P* < 0.001), while documentation of a new plan of management improved from 30 (60%) to 45 (90%) (χ² = 10.45, *P* = 0.001).

These statistically significant improvements across all measured parameters underscore the effectiveness of the structured documentation intervention. The introduction of a standardized proforma, combined with focused training and visual prompts, led to considerable enhancements in the completeness and consistency of follow-up notes. These findings, along with a detailed breakdown of frequencies, percentages, chi-square values, and *P*-values for each parameter, are presented in Table 2, clearly illustrating the magnitude of improvement achieved between the two audit cycles.

**Table 2:** Comparison of documentation quality in follow-up clinical notes before and after intervention at Hasahesa Hospital. This table presents the frequencies and corresponding percentages of compliance with 10 documentation parameters across two audit cycles (*n* = 50 per cycle). The first cycle represents baseline data collected in January 2025, and the second reflects post-intervention results from March 2025 following the introduction of a structured proforma and training. Improvements are shown as absolute percentage increases. Statistical significance for each parameter was assessed using the chi-square (χ²) test, with a *P*-value < 0.05 considered significant.

Parameter	First cycle (*n* = 50), *n* (%)	Second cycle (*n* = 50), *n* (%)	Improvement (%)	Chi-square (χ²)	*P*-value
Date and time	17 (34%)	45 (90%)	63%	30.94	<0.001
New chief complaint	15 (30%)	44 (88%)	60%	32.41	<0.001
History of present illness	15 (30%)	38 (76%)	45%	19.43	<0.001
Past medical history	10 (20%)	40 (80%)	60%	33.64	<0.001
Current medication	8 (16%)	40 (80%)	64%	38.50	<0.001
Vital signs	40 (80%)	49 (98%)	18%	6.54	0.011
Physical examination	27 (54%)	47 (94%)	40%	18.76	<0.001
Lab results	8 (16%)	45 (90%)	75%	52.03	<0.001
New provisional diagnosis	10 (20%)	45 (90%)	69%	46.71	<0.001
New plan of management	30 (60%)	45 (90%)	30%	10.45	0.001

## Discussion

The audit revealed a substantial improvement in the quality of follow-up medical documentation at Hasahesa Teaching Hospital, with total compliance rising from 38.2% in the first audit cycle to 87.2% post-intervention. This significant improvement demonstrates the efficacy of the actions enacted, which included the implementation of a standardized documentation template and focused staff training. The findings highlight the importance of organized documentation in supporting clinical accuracy, facilitating patient care, and enhancing institutional communication. However, further evaluation using direct clinical outcome measures such as complication rates or readmissions would be needed to confirm the impact of improved documentation on these areas.

Enhancements were seen in all assessed documentation criteria, favorably influencing both compliance rates and the wider dimensions of clinical practice and patient safety. These results align with prior research that recognized the advantages of organized formats compared to unstructured ones. One study reported that documentation quality increased from an average score of 64.35% to 77.2% following the implementation of standardized electronic templates, underscoring the critical importance of organized documentation in improving healthcare delivery [7]. Another investigation, which focused on manual/paper-based systems, revealed a 56% enhancement in compliance with NMBI documentation standards after introducing planned training initiatives alongside standardized SOAP note forms. Highlighting the type of system used in these studies allows better comparison with our findings and suggests that both electronic and paper-based structured formats can significantly improve documentation quality [8].

Documentation of the primary complaint significantly increased from 15 (30%) to 44 (88%) notes, reflecting a 60% improvement. This criterion is essential as it directs the doctor in formulating an appropriate treatment plan. The enhanced documentation probably indicates an increased recognition of its diagnostic significance. This discovery corresponds with the results of Shirazi et al., who highlighted the effectiveness of template-based documentation in reliably recording the patient's presenting issue [9]. Notable enhancements were documented in the history of current sickness and prior medical history, increasing by 45% and 60%, respectively. These areas are essential for constructing a comprehensive clinical overview and customizing personalized treatment strategies. Mazer et al. also emphasized the beneficial link between systematic recordkeeping and the quality of patient histories [10].

One of the most significant improvements was in the documentation of current drugs, which rose from 8 (16%) to 40 (80%) entries. The initial low rate highlighted a significant deficiency in continuity of treatment and possible pharmaceutical safety hazards. The increase after the intervention underscores the importance of standardized templates in documenting this critical characteristic. Precise medication recordkeeping is essential in averting adverse drug events, a significant contributor to avoidable morbidity and death in hospital environments [11].

The documentation of vital signs markedly improved, rising from 40 (80%) to 49 (98%) notes. Vital signs act as preliminary markers of patient decline and are crucial for prompt management. Previous research indicates that factors like respiratory rate are often overlooked, despite their significance in identifying early indicators of clinical deterioration [12]. This audit underscores the necessity for ongoing surveillance and documentation of these indicators. The documenting of physical examinations increased by 40%, increasing from 27 (54%) to 47 (94%) notes. Thorough physical exams are essential for precise diagnosis and continuous patient monitoring, and enhanced documentation likely indicates the efficacy of clinical training and awareness programs.

Documentation of lab results had a significant rise, escalating from 8 (16%) to 45 (90%) notes. This enhancement signifies improved incorporation of diagnostic data into therapeutic decision-making. Enhancements were seen in the documentation of new provisional diagnoses and treatment plans, increasing from 10 (20%) to 45 (90%) and from 30 (60%) to 45 (90%) notes, respectively. These elements are crucial for the continuity of treatment and accountability in medical planning.

The audit indicates that organized documentation tools and focused instructional interventions may significantly improve the completeness and correctness of clinical notes in resource-constrained, paper-based hospital environments. To maintain these advancements, it is essential to institutionalize these processes through ongoing education, regular audits, and the strengthening of documentation requirements. Future quality enhancement initiatives should not only target areas with initially poor adherence, such as medical history, prescription lists, and diagnostic planning, but also aim to evaluate how improvements in documentation directly affect patient care outcomes by incorporating relevant clinical metrics where feasible.

Limitations

This study has several limitations that should be considered when interpreting its findings. First, the relatively small sample size (*n *= 100) limits the generalizability of the results, especially in the absence of formal sample size calculation or power analysis. While the improvements observed were statistically significant, larger scale studies would be necessary to confirm the reproducibility of these findings across different settings and departments.

Second, the study did not explore potential implementation challenges associated with introducing structured documentation practices. Factors such as increased staff workload, time constraints, and logistical or technical barriers were not assessed, nor were the financial costs and resource implications of developing and disseminating structured templates and providing training. This was primarily due to the study’s focus on assessing documentation quality improvement within a limited timeframe and available resources. Evaluating these parameters would require a more comprehensive, mixed-methods approach, potentially involving qualitative data collection, which was beyond the scope of this audit. We recommend that future research investigate these important factors to better understand the feasibility and sustainability of such interventions.

Additionally, the perspectives and feedback of healthcare providers regarding the acceptability and usability of the new documentation system were not collected in this study. Future research should incorporate end-user feedback to better understand barriers and facilitators, which is essential for optimizing interventions and ensuring their sustainability.

Importantly, the audit focused exclusively on compliance with documentation standards and did not assess whether improved documentation translated into better clinical outcomes or patient safety. As a result, it remains unclear whether the enhancements in record-keeping reflect genuine improvements in the quality of care or merely procedural adherence.

## Conclusions

This research showed that standardized templates, staff training, and revised documentation processes improved follow-up clinical documentation at Hasahesa Teaching Hospital. Improvements in documentation of primary complaints, history of existing disease, current medications, and test findings indicate greater accuracy, consistency, and adherence to best practices. These findings support existing literature suggesting that organized documentation enhances the clarity, efficiency, and clinical value of medical records. However, the audit found gaps in prior medical history and provisional diagnostic documentation, underlining the need for ongoing education, quality monitoring, and established processes. The results demonstrate improvements in documentation quality through structured and detailed approaches. While these improvements have the potential to enhance patient safety, clinical decision-making, and healthcare system performance, especially in resource-limited settings, this study did not directly measure patient care outcomes. Further research is needed to confirm these effects.

## Appendices

Appendix 

**Table 3:** Follow-up Notes Documentation Checklist This checklist was used to evaluate the quality of follow-up note documentation in medical records. It comprises ten critical elements necessary for accurate and comprehensive patient documentation, including date and time, clinical findings, updated diagnoses, treatment modifications, medication lists, laboratory results, and follow-up instructions. Each element was assessed for its presence and completeness to determine overall documentation quality and adherence to best practices. Podder V, Lew V, Ghassemzadeh S: SOAP notes. StatPearls [Internet]. StatPearls Publishing, Treasure Island; 2023.04:43 PM

Checklist Item	Description
Date and Time	Documentation includes the exact date and time of the follow-up entry.
Clinical Findings	Relevant examination or clinical status is clearly stated.
Updated Diagnoses	Any changes or updates to the diagnosis are recorded.
Treatment Modifications	Notes include any changes in the treatment plan.
Medication List	An up-to-date list of current medications is documented.
Laboratory Results	Relevant test results are included or referenced.
Planned Follow-up Instructions	Clear instructions for follow-up care are provided.
Responsible Clinician	Name and designation of the documenting clinician are included.
Legibility	The note is readable (if handwritten) or clearly typed.
Completeness and Accuracy	Information is thorough and consistent with patient status and care provided.
